# An Adult Case of Severe Asymptomatic Bilateral Ureteropelvic Junction Obstruction

**DOI:** 10.1155/2023/9355564

**Published:** 2023-09-13

**Authors:** Md Fahad Hossain, Syed Sheheryar Shah, Bahar Bastani

**Affiliations:** Division of Nephrology, Department of Medicine, Saint Louis University School of Medicine, St. Louis, USA

## Abstract

While ureteropelvic junction (UPJ) obstruction is a well-recognized cause of unilateral or bilateral upper urinary tract dilatation in infants and the pediatric population, its occurrence in adults is less recognized. We present the case of a 68-year-old man who was being evaluated for chronic orthostatic hypotension and was incidentally found to have asymptomatic microscopic hematuria on urinalysis. A CT scan of the abdomen/pelvis, without and with contrast, revealed severe bilateral hydronephrosis due to UPJ obstruction. The patient has remained asymptomatic with preserved normal renal function over 7 years of follow-up.

## 1. Introduction

UPJ obstruction can be either congenital or acquired due to intrinsic or extrinsic factors. While in the pediatric age group, the most common cause of the dilated upper urinary tract is congenital UPJ obstruction (∼80% of fetal hydronephrosis cases), its incidence in adults is less well defined, though it is not a rarity. Although a significant number of these cases will eventually require surgical intervention, some patients will not present with functional obstruction till later adulthood. The stability in renal function depends on the anatomical cause and degree of UPJ obstruction, compliance of the renal pelvis, and urinary flow rate and output. We present an elderly male who was incidentally found to have asymptomatic severe bilateral UPJ obstruction with normal renal function.

## 2. Case Presentation

A 68-year-old man who was being evaluated for chronic orthostatic hypotension was incidentally found to have asymptomatic microscopic hematuria on a routine urinalysis.

**Figure 1:**
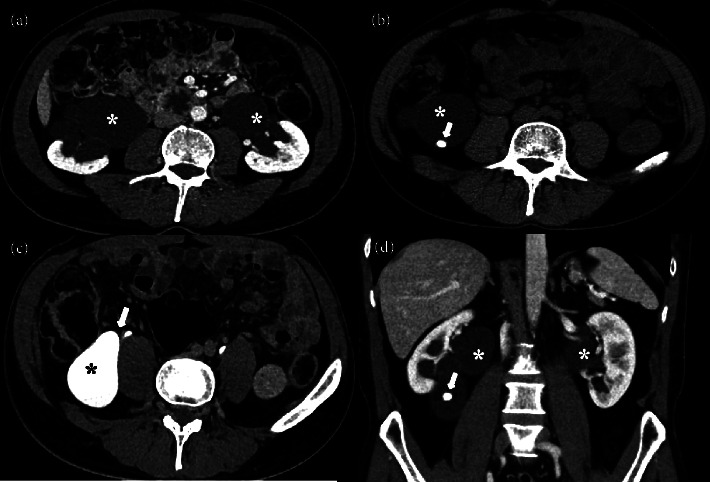
(a) The nephrogram phase of the contrast enhanced CT scan shows severe bilateral dilatation of the renal calyceal system and renal pelvises (^*∗*^). (b) The CT scan shows a severely dilated right kidney pelvis (^*∗*^) with a nonobstructing stone in it (arrow). (c) The urogram phase of the contrast enhanced CT scan shows a severely dilated contrast filled right renal pelvis (^*∗*^) and a normal caliber right renal ureter separated by ureteropelvic junction stricture (arrow). (d) The coronal view of the nephrogram phase of the contrast enhanced CT scan shows bilateral dilatation of renal pelvises (^*∗*^), more severe in the right side, and a nonobstructing stone in the right renal pelvic (arrow).

His renal function was normal with a stable serum creatinine level of 1.0–1.1 mg/dL (eGFR: ∼70 ml/min/1.73 m^2^) and no proteinuria. A CT scan of the abdomen/pelvis, without and with IV contrast, and CT urography (Figure 1) revealed severe bilateral ureteropelvic junction (UPJ) narrowing with severe bilateral hydronephrosis and multiple non obstructing stones in the right kidney. No aberrant crossing vessels were found. It also showed incidental segmental thickening of the terminal ileum which was later diagnosed as Crohn's disease. The furosemide washout MAG3-Tc^99^ the scan showed continuous radiotracer clearance from both kidneys, slower from the right kidney compared to the left. The split function was 53% left and 47% right.

Renogram peak time (min): 18 (left) and 27 (right); half-peak time: 47 (left) and 60 (right). Furosemide renogram half-peak time (min): 11 (left) and 10 (right).

He has been managed conservatively with periodic monitoring of renal function and imaging without any progression of kidney dysfunction over the past 7 years.

## 3. Discussion

Congenital UPJ obstruction occurs in 1 in 500 live births, is more common in males, and is more frequently on the left side [1]. Hydronephrosis is the most common mode of presentation. Other common clinical presentations are loin pain, abdominal mass, ureteric stone, hematuria, fever, nausea/vomiting, pyelonephritis, etc. The cases of UPJ obstruction can be either primary, where the actual pathophysiological cause is still unclear, or secondary, mainly due to extrinsic pressure [2, 3]. Some possible underlying pathophysiologic mechanisms have been abnormal arrangement of the smooth muscles of the proximal ureter resulting in an aperistaltic segment, high insertion of the ureter obliquely into the renal pelvis resulting in functional obstruction at UPJ when the renal pelvis is full, or crossing of the aberrant vessels to the lower pole of the kidney causing extrinsic pressure at UPJ [2, 4–6]. One study found that interstitial cells of Cajal (ICC), functioning as peristaltic pacemaker cells in UPJ, were less in number in patients with congenital UPJ obstruction compared to the normal controls [3]. Although there are also studies that found more ICC in UPJ, pooling data from different studies support the reduction of ICC theory [7]. Variations in the morphology of ICC have also been seen between UPJ obstruction patients and normal people [8]. Another study implicated immune mechanisms in the urothelium contributing to UPJ obstruction [9]. Moreover, in an endemic region, schistosomal fibrosis/stricture of UPJ was the second major cause of bilateral UPJ obstruction after congenital cases [10]. Although many cases with bilateral UPJ obstruction may progress with deterioration in kidney function, our patient despite having severe bilateral pelvicalyceal dilation has maintained stable renal function over the past several years, and the furosemide washout renogram showed clearance of the radiotracer from both renal pelvises. Indications for surgical intervention are intermittent or constant loin pain, acute pyelonephritis not responding to conventional treatment, worsening of renal function, gradual thinning of the renal cortex, and split renal function <40% in unilateral cases [2]. The Onen grading system is another objective way of determining the need for surgery [11].

## Data Availability

The deidentified data that support the findings of this case report are presented in the text.
